# Familiarity Detection is an Intrinsic Property of Cortical Microcircuits with Bidirectional Synaptic Plasticity

**DOI:** 10.1523/ENEURO.0361-16.2017

**Published:** 2017-05-22

**Authors:** Xiaoyu Zhang, Han Ju, Trevor B. Penney, Antonius M.J. VanDongen

**Affiliations:** 1 NUS Graduate School for Integrative Sciences and Engineering, National University of Singapore, 117456 Singapore; 2 Program for Neuroscience and Behavioral Disorders, Duke-NUS Medical School, 169857 Singapore; 3 Department of Psychology, National University of Singapore, 117570 Singapore

**Keywords:** familiarity, learning, NMDA receptor, plasticity, recognition memory

## Abstract

Humans instantly recognize a previously seen face as “familiar.” To deepen our understanding of familiarity-novelty detection, we simulated biologically plausible neural network models of generic cortical microcircuits consisting of spiking neurons with random recurrent synaptic connections. NMDA receptor (NMDAR)-dependent synaptic plasticity was implemented to allow for unsupervised learning and bidirectional modifications. Network spiking activity evoked by sensory inputs consisting of face images altered synaptic efficacy, which resulted in the network responding more strongly to a previously seen face than a novel face. Network size determined how many faces could be accurately recognized as familiar. When the simulated model became sufficiently complex in structure, multiple familiarity traces could be retained in the same network by forming partially-overlapping subnetworks that differ slightly from each other, thereby resulting in a high storage capacity. Fisher’s discriminant analysis was applied to identify critical neurons whose spiking activity predicted familiar input patterns. Intriguingly, as sensory exposure was prolonged, the selected critical neurons tended to appear at deeper layers of the network model, suggesting recruitment of additional circuits in the network for incremental information storage. We conclude that generic cortical microcircuits with bidirectional synaptic plasticity have an intrinsic ability to detect familiar inputs. This ability does not require a specialized wiring diagram or supervision and can therefore be expected to emerge naturally in developing cortical circuits.

## Significance Statement

Humans recognize familiar faces instantly. The cellular mechanisms underlying this recognition memory are still poorly understood. Simulations presented here demonstrate that bidirectional synaptic plasticity is sufficient to endow recurrent spiking neuronal network models with the ability to detect familiar sensory inputs through unsupervised learning. Network spiking activity evoked by a face image results in changes in synaptic connectivity and the formation of a unique strengthened subnetwork. Networks can recognize multiple previously seen faces with high accuracy by forming partially overlapping subnetworks. We therefore propose that familiarity detection is an intrinsic property of generic cortical microcircuits with bidirectional synaptic plasticity.

## Introduction

Recognition memory refers to the ability to recognize previously experienced sensory inputs. Prior studies (Nickerson, 1965; Shepard, 1967) found that immediately following a single exposure to 612 pictures, subjects could select the previously-seen picture in two-alternative recognition tests with 98% accuracy. Later, Standing (1973) reported that the number of pictures correctly recognized increases with the number of pictures presented (up to 10,000), suggesting a limitless capacity of recognition memory. These experimental observations can be accounted for by familiarity, a form of unsupervised learning. Familiarity and recollection are two major processes that underlie recognition memory (Yonelinas, 2002; Squire et al., 2007). While recollection demands accurate recall of the object’s features, familiarity merely requires a signal indicating that the object has been previously encountered (Wixted, 2007). Evidence from functional imaging studies shows that cortical regions surrounding the hippocampus become active when a human subject senses a familiar input, whereas activation of the hippocampus is required for recollection (Hölscher et al., 2003; Diana et al., 2007).

While learning and memory have been extensively studied at the molecular/cellular (Wu et al., 2006; Baudry et al., 2015) and behavioral (Gale et al., 2014; Rapanelli et al., 2015) levels, it has been difficult to causally connect these two levels of desciption (Morgado-Bernal, 2011). Memories are believed to be encoded by and stored in a subset of neurons (the “engram”), which are connected by synapses whose weights were altered by the learning experience (Takeuchi et al., 2014). Given the current knowledge on how learning induces changes in synaptic efficacy through long-term potentiation (LTP; Bliss and Lomo, 1973), long-term depression (LTD; Ito and Kano, 1982; Massey and Bashir, 2007), and spike-timing-dependent plasticity (STDP; Gerstner et al., 1996; Markram et al., 1997; Bi and Poo, 1998), it is meaningful to simulate the synaptic changes in biologically plausible neural networks, to foster understanding from a systems perspective.

The liquid state machine (LSM) is a biologically-inspired spiking neural network model, which closely emulates the complexity of a generic cortical microcircuit. It is designed to perform biologically realistic real-time computing on time-varying inputs, providing an alternative to the widely-used attractor neural networks which require convergence to stable internal states (Maass et al., 2002). The construction of an LSM network, which involves generating random connections with random synaptic weights, is task independent. An important property of these networks is that sensory inputs are expanded into high-dimensional feature space, allowing linear separation of complex properties (Buonomano and Maass, 2009). Temporal and spatial input information is transiently preserved in the form of fading memory, through multiple recurrent loops and short-term synaptic plasticity (depression and facilitation). We have introduced activity-dependent long-term synaptic plasticity into the network model by incorporating NMDA receptor (NMDAR) functionality (Shouval et al., 2002) in the excitatory synapses.

NMDARs are critically important for synaptic plasticity-dependent learning. When post-synaptic depolarization coincides with glutamate and glycine binding, NMDARs open and allow Ca^2+^ influx (Blanke and VanDongen, 2009). Depending on the amount of Ca^2+^ influx, it will selectively activate phosphatases (low calcium influx) or the kinase CaMKII (high calcium influx), and trigger downstream signaling for synaptic depression or potentiation, respectively (Salter et al., 2009; Malleret et al., 2010; Luscher and Malenka, 2012), a theory known as the calcium control hypothesis (Lisman, 1989). LTP and LTD can be induced by tetanic synaptic inputs that regulate the Ca^2+^ influx. They are rate-based: low-frequency stimulation causes LTD, while a high-frequency tetanus induces LTP. STDP is another form of synaptic plasticity regulated by the temporal correlation of pre- and post-synaptic firing. By implementing back-propagating action potentials (BPAPs), NMDAR functionality can support STDP through calcium control (Waters et al., 2005; Paradiso and Wu, 2009). We have implemented both rate-based (LTP/LTD) and spike-timing-based (STDP) plasticity, by modeling NMDAR functionality in the excitatory synapses using the calcium control hypothesis.

The NMDAR-containing neural network is a model of generic cortical microcircuits with the capability of unsupervised learning. We have used it here to study how familiarity could develop in the cortex. On a large scale, brain regions are wired into relatively deterministic neural circuits (Coutlee and Huettel, 2012; Fornito et al., 2012), yet randomness and flexibility prevails within local cortical regions, with functional connections being optimized by activity-dependent changes (Sporns and Zwi, 2004; Fair et al., 2009). The main hypothesis underlying our simulations is that a sensory stimulus induces changes in synaptic weights in a neural microcircuit, altering the network response such that it can distinguish familiar from novel inputs.

## Materials and Methods

### Neural network implementation

Neural networks were simulated using MATLAB and the CSIM package (a neural Circuit SIMulator, RRID: SCR_014256, available at http://www.lsm.tugraz.at/csim/), as described previously (Natschläger et al., 2002; Ju et al., 2013). NMDAR-dependent synaptic plasticity was introduced into the excitatory synapses following the model proposed by Shouval et al. (2002). The neural networks consist of two parts: an input layer, and the network reservoir. Input neurons send spikes to the network reservoir via static spiking synapses, which have no plasticity. The network reservoir consists of leaky integrate-and-fire (LIF) neurons recurrently connected by NMDAR synapses. Seventy-five percent of the neurons are set as excitatory, the remaining being inhibitory. Each LIF neuron is modeled by a linear differential equation:

(1) $${\text{τ}}_{m}\frac{{\text{dV}}_{m}}{\text{dt}}\;=-\left({\text{V}}_{m}–{\text{ V}}_{\text{resting}}\right)+{\text{ R}}_{m}\left({\text{I}}_{\text{syn}}+{\text{ I}}_{\text{inject}}+{\text{I}}_{\text{noise}}\right),$$

where the parameters are: membrane time constant τ_m_ = 30 ms, V_resting_ = 0 mV, membrane resistance R_m_ = 1 MΩ, input currents supplied by explicitly modeled synapses I_syn_, steady background current I_inject_ = 13.5 nA and for some simulations random noise I_noise_ was added to the current. For the first time step in the simulation, the membrane potential V_m_ was set to an initial random value between −1 and 1 mV. When V_m_ increases to 15 mV (the firing threshold), the neuron fires, and V_m_ is reset to a random value between −1 and 1 mV after an absolute refractory period of 3 ms for excitatory neurons and 2 ms for inhibitory neurons (Joshi, 2007).

In CSIM, the probability that two neurons are connected by a synapse is defined as:

(2) $$P\left(D\right)=C\cdot \exp\left(\frac{-D^{2}\left(a,b\right)}{\lambda^{2}}\right),$$

where *D(a,b)* stands for the Euclidean distance between the two neurons a and b. λ and C are parameters used by CSIM that determine connectivity and synaptic strength, respectively. As λ increases, both the connection probability and the average connection length will increase. The base value of C depends on the type of connection: it is set to 0.3, 0.2, 0.4, and 0.1 for EE, EI IE, and II connections, where E and I stand for excitatory and inhibitory neurons. The values are based on recordings from rodent cortical brain areas (Gupta et al., 2000). The actual value of C is modulated by a user-defined parameter, Cscale. Input layer neurons are all excitatory. Connections from input neurons to the network reservoir and within the network reservoir are randomly generated following the probability *P(D)*.

Once a connection is established, it is assigned an initial synaptic weight, indicating synaptic efficacy. Initial synaptic weights are drawn from the following gamma distribution:

(3) $$y=f(x|a,b)=\frac{1}{b^{a}\text{Γ}\left(a\right)}x^{a-1}e^{\frac{-x}{b}}a=\frac{1}{SH\_W^{2}},b=W\cdot SH\_W^{2},$$

where *Γ (·)* is the Gamma function. *SH_W* and *W* are parameters used by CSIM. *SH_W* (default 0.7) positively correlates with the variance of the weight distribution and *W* correlates with the mean of the distribution. The base value of *W* is set to 3e^−8^ for EE, 6e^−8^ for EI, −1.9e^−8^ for IE and II. The actual value of *W* is modulated by a user defined parameter, Wscale. The synaptic weight of an excitatory NMDAR synapse is subject to strengthening (upper boundary = 6.5e^−8^) or weakening (lower boundary = 1.0e^−9^) by plasticity. Synapses from the inhibitory neurons have negative weights, and do not possess plasticity. Synaptic weights of the static spiking synapses from input neurons are fixed.

**Figure 1.**
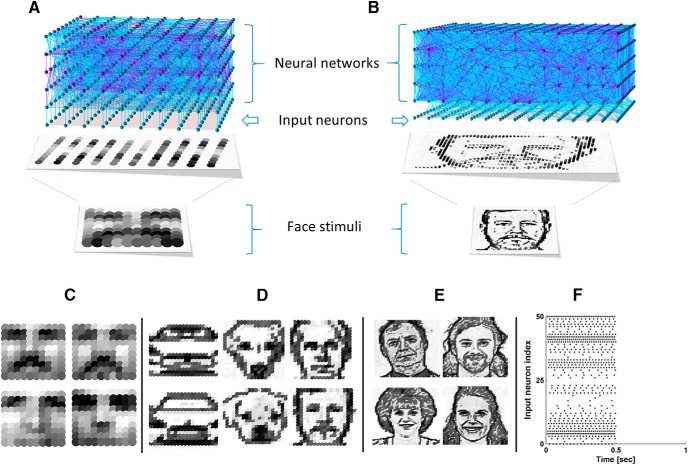
Network architecture and stimulus encoding. ***A***, Diagram illustrating a 10 × 10 × 5 network with a 10 × 10 input layer, receiving a stimulus from a beard face image (10 × 10 pixels). Neurons in the network reservoir are located at positions with integer coordinates in a 3-D space. The input layer is located 1 unit away from the network reservoir. Input neurons form synapses one-to-one with the first layer of the network reservoir. Each input neuron receives a spike train with a firing rate determined by the corresponding image pixel intensity value. ***B***, Diagram illustrating a 50 × 50 × 5 network (only a portion of size 15 × 15 × 5 is shown) with a 50 × 50 input layer (only 15 × 15 portion is shown), receiving a stimulus from a human face image (50 × 50 pixels). Input neurons form synapses with random neurons in the network reservoir. ***C***, Examples of a 10 × 10-pixel beard face (top) and a 10 × 10-pixel no-beard face (bottom). ***D***, Examples of 20 × 20-pixel images of car fronts, dog faces and human faces. ***E***, Examples of 50 × 50-pixel human faces. ***F***, A sample beard stimulus which contains spike trains (0–0.5 s), followed by a silent interval (0.5–1.0 s). Only 50 channels of spike trains are shown for clarity.

In CSIM, a network is generated by placing neurons on a 3-D grid. The networks described in Figure 1*A* had five layers with 10 × 10 neurons each (dimension, 10 × 10 × 5). Input neurons formed synapses one-to-one with the first layer of the network reservoir, with fixed synaptic weights of 2.7e^−7^. NMDAR synapses in the network reservoir were generated with λ = 2.0 and Cscale = 1.0. Initial weights followed the gamma distribution with SH_W = 0.25 and Wscale = 0.5.

Networks described in Figure 1*B* consisted of five or six layers, with dimensions 20 × 20 × 5, 50 × 50 × 5, and 50 × 50 × 6. In this case, input neurons formed synapses randomly with the network reservoir with Cscale = 0.04, 0.004, and 0.005, respectively, and λ was set to infinity in all cases to remove the limitation by distance. As a result, there was no topographical mapping of the input pattern. Input synaptic weights were still fixed but no longer uniform, following a gamma distribution (Wscale = 3, SH_W = 0.7 in all cases) instead. As for NMDAR synapses in the network reservoir, λ = 4.0 for the 20 × 20 × 5 networks; λ = 3.0 for 50 × 50 × 5 and 50 × 50 × 6 networks; Cscale = 1 for all cases. The λ values were chosen to make sure that each neuron formed ∼100 synapses on average with others in the network. Initial weights of NMDAR synapses also followed a gamma distribution with Wscale = 0.9 and SH_W = 0.25. By setting Wscale to 0.9, the initial weights were set to intermediate values, leaving enough room for future potentiation and depression. By setting SH_W to 0.25 for the network reservoir, we reduced the variation in the initial weights, thereby reducing any preimposed network circuitry.

### Synaptic plasticity implementation

The NMDAR-dependent plasticity we implement follows the model by Shouval et al. (2002). Synaptic plasticity (LTP/LTD and STDP) depends critically on the amplitude and timing of postsynaptic EPSPs and BPAPs. BPAPs were not implemented in the original CSIM, while EPSPs were implemented using a single exponential decay function with a time constant of 3 ms. We introduced BPAPs and changed both BPAPs and EPSPs to follow double-exponential decays, each with a fast and a slow component. Decay time constants were scaled from the suggested values (Shouval et al., 2002), with BPAP fast decay time constant $\tau_{f}^{bs}$ = 1.2 ms and proportion $I_{f}^{bs}$ = 0.75, slow decay time constant $\tau_{s}^{bs}$ = 10 ms and proportion $I_{s}^{bs}$ = 0.25; EPSP fast decay time constant $\tau_{f}^{ep}$ = 2 ms, proportion $I_{f}^{ep}$ = 0.5, slow decay time constant $\tau_{s}^{ep}=$ 20 ms and proportion $I_{f}^{ep}$ = 0.5.

(4) $$BPAP\left(t\right)=BPAP\_max\cdot (I_{f}^{bs}e^{-t/{\text{τ}}_{f}^{bs}}+I_{s}^{bs}e^{-t/{\text{τ}}_{s}^{bs}})$$

(5) $$EPSP\left(t\right)=EPSP\_max\cdot (I_{f}^{ep}e^{-t/{\text{τ}}_{f}^{ep}}+I_{s}^{ep}e^{-t/{\text{τ}}_{s}^{ep}})$$

Using a double-exponential decay ensures that the BPAP has a sharp peak with a thin tail and that the EPSP has a slower peak and more prominent tail, thereby preserving the difference in temporal signature between EPSPs and BPAPs. This contrast ensures that the change in intracellular Ca^2+^ concentration induced by pre-post firing exceeds the [Ca^2+^] change induced by post-pre firing, so that STDP will be induced properly (see Fig. 2 in Shouval et al., 2002).

**Figure 2.**
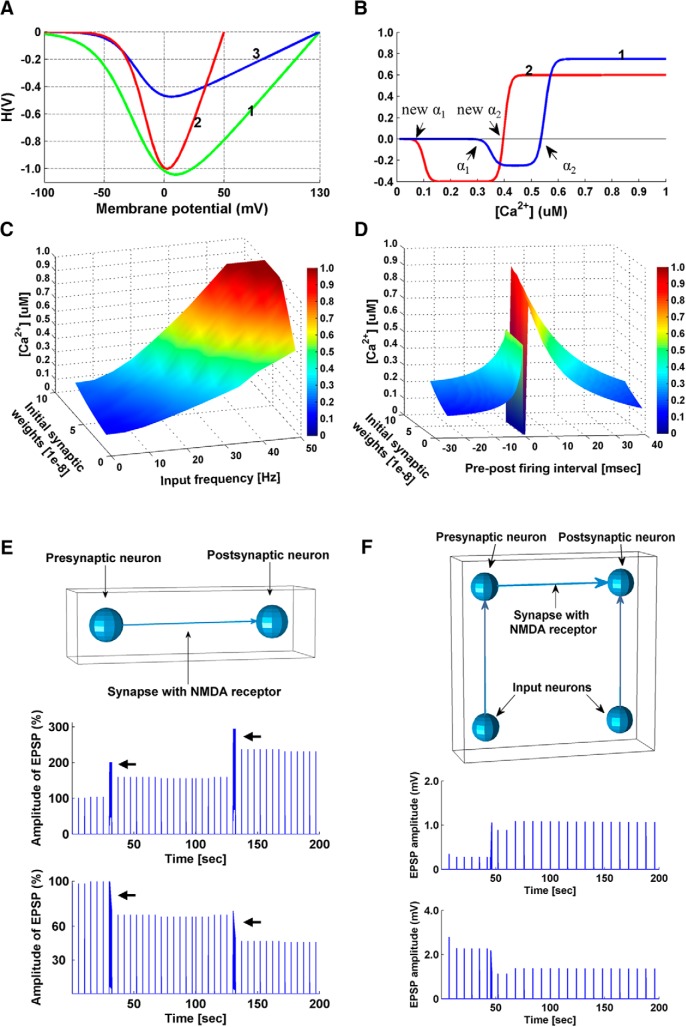
Parameter turning. ***A–D***, Calcium control hypothesis. *A*, Curves illustrating the driving force function H(V): (1) as proposed in (Shouval et al., 2002); (2) from *in vitro* recordings of ion currents through NMDARs (Pattillo et al., 1999); (3) re-parameterized in our model. Curve (3) is similar to (2) at negative membrane potentials. Curve (3) has the same equilibrium potential as (1) but is smaller in amplitude, reducing the impact of growing synaptic weights. ***B***, Models of the calcium control hypothesis Ω function: (1) as proposed in (Shouval et al., 2002), (2) as used in our simulations. When real-time synaptic Ca^2+^ concentration ([Ca^2+^]) exceeds the new α_1_ but not the new α_2_, synaptic weight is decreased; when real-time synaptic [Ca^2+^] exceeds the new α_2_, synaptic weight is increased. ***C***, Real-time postsynaptic [Ca^2+^] is plotted as a function of input frequency and initial synaptic weights. ***D***, Real-time postsynaptic [Ca^2+^] is plotted as a function of pre-post firing intervals and initial synaptic weights. ***E***, Induction of LTP/LTD at individual synapses following high/low-frequency stimulation protocols. Top, Model setup. A presynaptic input neuron is connected to a postsynaptic neuron by an excitatory NMDAR synapse. The presynaptic input neuron generated spikes at a certain frequency, and EPSPs were recorded in the postsynaptic neuron. The postsynaptic neuron is prevented from firing action potentials. Middle, Simulation results with 50 Hz tetanus input on the model. Spike trains of 50 Hz were generated by the presynaptic input neuron, which caused long-lasting increases of the EPSP amplitude in the postsynaptic neuron. Arrows indicate when a 50 Hz tetanus (2 s) was applied. Bottom, Simulation results with 20 Hz tetanus input on the model, which resulted in a long-lasting decrease in the amplitude of EPSPs. Arrows indicate when a 20 Hz tetanus (2 s) was applied. Results are comparable to observations from Bliss and Lomo (1973) and Ito and Kano (1982). Baseline spikes at a frequency of 0.2 Hz were generated by the presynaptic input neuron, in addition to the tetani, to probe the changes in EPSP amplitude. ***F***, Simulation of a dual patch clamp STDP experiment. Top, Model setup. A presynaptic neuron is connected to a postsynaptic neuron by an excitatory NMDAR synapse. EPSPs were recorded in the postsynaptic neuron. Two input neurons are connected to the pre- and post-synaptic neurons to control their firing time. STDP simulation results are comparable to observations of STDP in cortical neurons (Froemke et al., 2010). Middle, Potentiation was induced in the model by pre-post firing (Δ*t* = 15 ms, 15 pairings). Bottom, Depression was induced by post-pre pairing (Δ*t* = −75 ms, 30 pairings). Baseline spikes were generated at a frequency of 0.125 Hz by the input neuron connected to the presynaptic neuron to probe the changes in EPSP amplitude.

Activity-induced EPSPs generate the driving force for calcium currents through the NMDAR:

(6) $$H(V)=\frac{-0.42(\text{V}-{\text{V}}_{r})}{\left(1+0.6e^{-0.09\text{V}}\cdot [{\text{Mg}}^{\text{2+}}]/3.57\right)}$$

where *V* = *V*_resting_ + EPSP + BPAP, and *V_r_*is the reversal membrane potential for calcium (130 mV). The Mg^2+^ concentration is set to 1 mM. The driving force function was modified from the function suggested by Shouval et al. (2002) which did not take into consideration the effect of growing synaptic weights. *In vitro* recordings of ion currents through NMDARs (Pattillo et al., 1999) show a steeper curve, and based on this, we re-parameterized the calcium driving force (Fig. 2*A*).

The calcium current through NMDARs ($I_{NMDA}$) is assumed to have this form:

(7) $$I_{NMDA}\left(t_{i}\right)=P_{0}G_{NMDA}[I_{f}\theta (t)e^{-t/{\text{τ}}_{f}}+I_{s}\theta (t)e^{-t/{\text{τ}}_{s}}]H(V)$$

where P_O_ is the probability of opening, G is the conductance of the NMDAR channel, *I*_f_ and *I*_s_ are the fast and slow components of the NMDA currents, τ_f_ and τ_s_ are the time constants for the fast and slow components.

### Calcium control hypothesis

The activity-dependent change in synaptic calcium concentration is modeled as a function of the NMDA current $I_{NMDA}$:

(8) $$\frac{dCa(t)}{dt}=I_{NMDA}\left(t\right)-(1/{\text{τ}}_{Ca})[Ca(t)]$$

where [Ca(t)] is the calcium concentration at the synapse at time *t* and $\tau_{Ca}$ is the decay time constant, which is assumed to be 50 ms. The weights of NMDAR synapses are modified by calcium concentration as follows:

(9) $$\dot{W_{j}}=\text{η}\left({\left[Ca\right]}_{j}\right)\left(\Omega ({\left[Ca\right]}_{j}\right)-W_{j})$$

The Ω function represents the calcium control hypothesis with α_1_ and α_2_ being [Ca^2+^] thresholds for depression and potentiation induction:

(10) $$\text{Ω}=\text{sig}\left(\left[Ca^{2+}\right]-{\text{α}}_{2},{\text{β}}_{2}\right)-\Omega_{rate}\cdot sig\left(\left[Ca^{2+}\right]-{\text{α}}_{1},{\text{β}}_{1}\right),where\,\;sig\left(x,\text{β}\right)=\frac{e^{\text{βx}}}{1+e^{\text{βx}}}$$

The parameter values were originally set as follows: α_1_ = 0.35 µM, α_2_ = 0.55 µM, β_1_ = β_2_ = 80 µM, and $\Omega_{rate}$ = 0.25 (Shouval et al., 2002). By setting new α_1_ to 0.1 µM and new α_2_ to 0.4 µM (Fig. 2*B*), we were able to induce LTD at low input frequency (1–20 Hz) and post-pre firing (−15 ∼ −5 ms) regardless of the synaptic weights, while LTP was obtained at high input frequency (50–100 Hz) and pre-post firing at (5 ∼ 15 ms), regardless of the synaptic weights (Fig. 2*C*,*D*; Table 1). We also changed $\Omega_{rate}$ from 0.25 to 0.4, rendering the plasticity more biased toward LTD to stabilize network activity after prolonged sensory exposure.

**Table 1.** Postsynaptic Ca^2+^ concentration [µM]

Synaptic weight	Input frequency [Hz]					Pre-post firing interval [msec]			
	1	10	20	50	100	−15	−5	5	15
1e^−8^ (small)	0.11	0.14	0.22	0.60	1.0	0.12	0.16	0.69	0.40
3e^−8^ (medium)	0.15	0.19	0.30	0.85	1.0	0.17	0.21	0.78	0.46
6e^−8^ (large)	0.27	0.32	0.52	0.90	1.0	0.29	0.33	0.93	0.61

Points are extracted from Figure 2C,*D*, showing [Ca^2+^] under input stimuli of frequency 1, 10, 20, 50, and 100 Hz, or pre-post firing intervals of −15, −5, 5, and 15 ms, when initial synaptic weights are small (1e^−^^8^), intermediate (3e^−^^8^), and large (6e^−^^8^). Red indicates LTP and blue indicates LTD.

### Individual synapse performance

After parameter tuning described in the sections above, we simulated the classic tetanus experiment (Bliss and Lomo, 1973) and dual patch clamping experiment (Markram et al., 1997) on single NMDAR synapses. The synaptic efficacy change successfully reproduced previously published results (Fig. 2*E*,*F*), indicating the reliability of our model at individual synpase level.

### Image preprocessing

For the beard versus no-beard classification simulations, grayscale images of male faces with and without a beard were selected to form two groups, 20 images for each group. Each image was reduced to a 10 × 10 pixel pattern (Fig. 1*C*). For the single face recognition simulations, we used grayscale images of car fronts, dog faces and human faces, 10 images for each category. Each image was converted to a line drawing using edge detection to reduce noise from shading and then down-sampled to a 20 × 20 pixel pattern (Fig. 1*D*). For the multi-face recognition simulations, 200 grayscale human face images were selected and each face image was converted to a line drawing and down-sampled to 50 × 50 pixels (Fig. 1*E*). Images were presented to the network by assigning each pixel to a corresponding input neuron and converting pixel values to spike trains. Grayscale values of 0–255 were mapped to frequencies of 0–50 Hz. Each image stimulus lasted 0.5 s, followed by a silent interval of 0.5 s (Fig. 1*F*) to allow the fading memory to dissipate and prepare the network for the next stimulus.

### Fisher’s discriminant analysis

Multiclass Fisher’s discriminant analysis was applied to network spiking activity and neuronal spiking activity, to obtain Fisher’s linear discriminant ratio (FDR). The FDR is calculated as follows:

(11) $$J\left(t\right)=\frac{\sum_{C}{\left(\mu_{C}\left(t\right)-\mu \left(t\right)\right)}^{2}}{\sum_{C}\sum_{i\epsilon C}{\left(S_{i}\left(t\right)-\mu_{C}\left(t\right)\right)}^{2}}$$

where $S_{i}\left(t\right)$ is the spike count at time bin t, in response to stimulus i; $\mu_{C}\left(t\right)$ is the mean spike count at time t for class C stimuli, and $\mu \left(t\right)$ is the mean of the class means $\mu_{C}\left(t\right)$. The numerator and denominator of the discriminant ratio J are known as the “between classes scatter” and “within class scatter.” A larger value of J indicates better discrimination. More information can be found in Ju et al. (2015).

Network FDR was obtained by summing up the FDRs calculated with network spiking activity in each time bin, serving as an indicator of network separability for input stimuli. Neuronal FDR was calculated with the spiking activity of individual neurons over the entire recording period, serving as an indicator of how informative the neuron is for input discrimination.

## Results

We performed simulations of cortical microcircuits modeled with NMDAR-containing neural networks (see Materials and Methods), to investigate whether they can develop recognition memory. Images were used as sensory inputs and plasticity was enabled during learning and disabled during evaluation of responses to the input images. Simulations typically consisted of three phases: baseline recording, learning and testing. First, to establish a baseline, network responses to all input stimuli were recorded while NMDAR function was disabled. Network firing rate (spikes/second) during the stimulus presentation was calculated and used as a measure of network response. In the second phase, learning was switched on by enabling NMDAR plasticity, and networks were stimulated using only a subset of images, allowing stimulus-induced network spiking activity to alter synaptic strengths. These images should now be “familiar” to the networks. Finally, learning was switched off and network responses to all input stimuli were recorded again. The effect of the learning experience was evaluated for both familiar images presented during the learning phase and the “novel” images, by comparing testing responses with their corresponding baseline values. In all of our simulations, we found that the networks responded differentially to images presented during the learning phase.

### Unsupervised classification: beard versus no-beard

**Figure 3.**
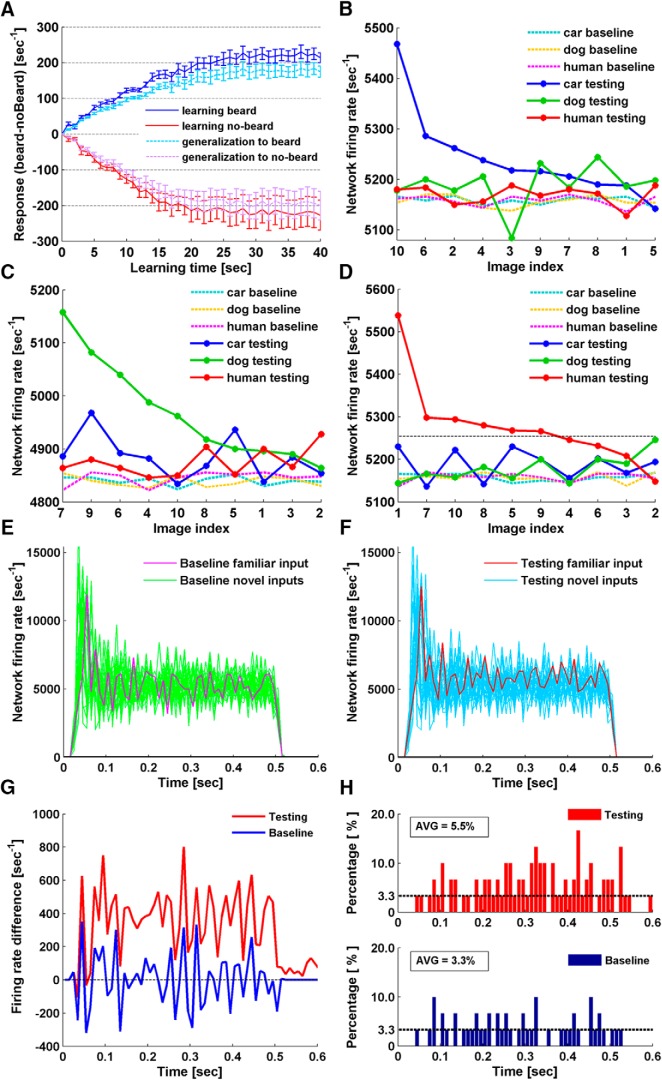
Unsupervised familiarity detection. ***A***, Beard versus no-beard classification. Curves showing the discrepancy in network responses (firing rate [sec^−1^]) to beard and no-beard faces that develops with sensory exposure to one class. The blue curves represent network response (beard-noBeard) after learning beard faces. The dark blue solid line shows response differences when trained beard faces were used for testing. The light blue dashed line shows the results when untrained beard faces were used for testing, i.e., networks generalizing familiarity to novel beard faces. The red curves represent the results from the same simulation paradigm with no-beard faces used for learning. The red solid line shows response differences when trained no-beard faces were used for testing. The pink dashed line shows the results when untrained no-beard faces were used for testing, i.e., networks generalizing familiarity to novel no-beard faces. Error bars at each time point reflect the SEM of results from five randomly-generated networks. Each network underwent five trials of simulation with randomly-selected face inputs. Curves were normalized by subtracting the discrepancy of network baseline response to the two classes at time 0. ***B–D***, Single face recognition. ***B***, The network test responses (solid lines) and corresponding baseline responses (dashed lines) to all 30 images after learning car image 10. Test responses to car images were sorted in descending order. As images of each class were indexed as 1–10, test responses to the other two classes are plotted following the sorted index order. ***C***, Network responses to all 30 images after learning dog image 7. ***D***, Network responses to all 30 images after learning human face 1. ***E***, ***F***, Firing rate dynamics during stimulus presentation of the network shown in ***D***. ***E***, Network baseline firing rate to all 30 stimuli. ***F***, Network firing rate to all 30 stimuli after learning human face 1. The stimulus of human face 1 is referred as the familiar input. ***G***, Firing rate difference between response to the familiar input and the average of the responses to the novel inputs. The average performance of 10 networks in 30 simulations has been shown. Blue represents the difference in network baseline responses. Red represents the difference in network testing responses, after the single face recognition simulations. ***H***, Histograms of time bins where the networks exhibited the highest firing rate to the familiar input. Network firing rate was obtained as in ***E***, ***F***, binning was at 10 ms. Time bins where networks exhibited the highest firing rate to the familiar input were identified. The number of the identified time bins was summed for 30 simulations carried out 10 networks, and the percentage was calculated with respect to 30 simulations, with chance level being 1/30 (3.3%).

In this first set of simulations, two classes of images were used: human faces of males with and without a beard. In each simulation, only faces from a single class (beard or no-beard) were presented to a network during the learning phase. In each learning round, one face from the selected class was randomly chosen and presented to the network for 1 s. NMDAR plasticity was switched off for testing after each round, and network responses to all faces from both classes were recorded. Figure 3*A* summarizes the performance of five randomly-generated 10 × 10 × 5 networks for 40 rounds of image presentation. Clearly, networks responded more strongly to faces from the trained class, and the firing rate discrepancy for the two classes increased as more faces were presented. In other words, networks started to display a differential response to the two classes as unsupervised learning took place. This was true whether the beard or no-beard class was used for training. The differential response eventually reaches a steady state, in part because we have set a maximum weight boundary (6.5e^−8^) for excitatory synapses to prevent overtraining. We also investigated network performance by excluding faces that were presented in the learning phase from the testing. The results showed a similar increase in discrepancy for the responses to the two classes, only with a reduced magnitude (Fig. 3*A*). In this case, the networks were performing a binary classification task, following unsupervised learning.

### Single face recognition

Next, we performed single face recognition simulations, for which we increased the dimension of the networks to 20 × 20 × 5 and the input layer size to 20 × 20. A set of 30 images was used as input stimuli, consisting of 10 car fronts, 10 dog faces, and 10 human faces. The simulations again consisted of three phases: baseline normalization, learning, and testing. NMDAR-dependent plasticity was only switched on during the learning phase. Normalization was performed by recursively adjusting the mean pixel value for each of the 30 images until they evoked comparable baseline responses. In the learning phase, a single human face image was selected from the set of 10, and presented repetitively for 15 s. NMDAR-dependent plasticity was then switched off for the testing phase, in which we recorded network responses to all images, to determine whether the network responded differently to the selected (learned) face.

For this simulation, we used 30 randomly-generated networks. Instead of limiting the stimuli to human faces, we also conducted trials using images of car fronts and dog faces. For each network, three trials were conducted, each with a randomly selected car, dog, or human image. In total, 90 trials were performed on the 30 networks, and in 84 cases (93.3%), the networks exhibited the highest firing rate to the image presented during the learning phase. Examples of network responses before and after the learning phase are shown in Figure 3*B–D*. Testing responses are sorted by firing rate and shown as solid lines, while corresponding baseline responses are plotted as dashed lines. Network firing rate to the images selected for learning significantly increased and became the largest on testing. Responses to novel inputs from the same class as the image selected for learning tended to be elevated from their baseline, although to a lesser extent than the actual learned image. For instance, in the human face learning simulation (Fig. 3*D*), the network responses to 5 of the 10 human faces (excluding the learned face) were higher than the responses to the dog and car images, suggesting that the network had also generalized to distinguish human faces from dog faces and car fronts. Considering the degree of randomness involved in network construction, and the similarity of the images within each class, we conclude that networks of 20 × 20 × 5 neurons can learn to detect a familiar image with high specificity.

The network responses in Figure 3*B–D* are network firing rates averaged over the entire recording period. In fact, the dynamic network firing rates plotted as a function of time underwent initial rising phases and declined to steady state phases (Fig. 3*E*,*F*). By solely looking at firing rate difference, network response to the familiar input seems to have increased over the entire recording period after learning (Fig. 3*G*). Whether the increase in individual time bins is sufficient for familiarity detection is further analyzed. If we assume the networks are able to detect familiarity in the time bins where networks exhibited the largest firing rate to the familiar input, we can count the number of such time bins and compare it before and after learning. The results show that there is no specific time window that clearly separates the familiar input from the novel inputs, but the separation gets better in the later stage of stimulus presentation (Fig. 3*H*).

### Multi-face recognition

Now that these relatively small neural networks have demonstrated they can discriminate a single familiar face from many novel ones, we tested whether networks can perform familiarity detection to more than one face. We began with presenting two human face stimuli to a single network, using the same set of 30 images and 20 × 20 × 5 networks. Unlike the high accuracy (93.3%) of the single face recognition simulations, familiarity detection accuracy for two face stimuli dropped to 70.0% (data not shown). This performance decline informs us of the limited capacity of 20 × 20 × 5 networks.

**Figure 4.**
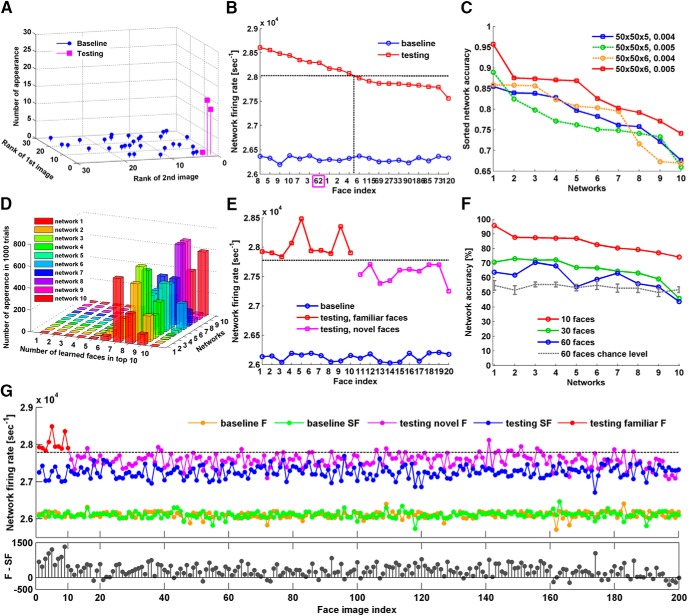
Familiarity detection for multiple faces. ***A***, Results of 50 × 50 × 5 networks learning two human faces. Each bar is located at the point whose coordinates represent the rank of network firing rate for the two learned faces. The height of each bar reflects the number of occurrences of such a combination (30 in total). Blue and magenta represent network baseline and testing response, respectively. Note that after learning, the two familiar faces ranked 1 and 2 in 29 of 30 trials and 1 and 3 in the remaining trial. ***B–E***, Results of learning 10 faces and testing them against 10 novel faces randomly drawn from a database of 200 faces. ***B***, Results of a 50 × 50 × 5 network learning 10 faces. The curves show baseline and sorted testing response after learning faces 1–10. The network ranks nine familiar faces among the top 10, with only one outlier (face 62, highlighted in magenta). The accuracy of this network therefore was 9/10 or 90%. ***C***, The effect of network dimension and Cscale value was evaluated by performing 1000 trials for 10 different networks (see Results). For each trial, the accuracy was calculated as in ***B***. The curves illustrate the accuracy (averaged over 1000 trials) for the 10 networks with dimensions and Cscale values indicated in the legend. ***D***, Accuracy distribution of 1000 testing trials conducted on the ten 50 × 50 × 6 networks with Cscale 0.005. ***E***, Baseline and testing response of the best-performing 50 × 50 × 6 network after learning face 1–10. Network firing rate differentiates familiar faces completely from novel faces. ***F***, Network average accuracy of the 50 × 50 × 6 networks after learning 10 faces (red) and the corresponding accuracy after learning 30 faces (green), 60 faces (blue), and chance level at 60 faces (gray, dotted line). Error bars are the SDs of 1000 trials. ***G***, top, Baseline and testing response of the best-performing 50 × 50 × 6 network to all 200 faces and their scrambled versions, after learning face 1–10. F, faces; SF, scrambled faces. Bottom, Pairwise network firing rate difference to faces and scrambled faces (F-SF).

We therefore increased dimensions of the networks to 50 × 50 × 5 and input layers size to 50 × 50. In addition, the resolution of the 30 images was increased to 50 × 50 pixels, so that their dimension matched that of the input layer. With these larger networks, the accuracy of familiarity detection for two face stimuli increased to 96.7%. The performance improvement could be due to either the increase in network size or the increase in resolution of the face images. To distinguish between these two options, we repeated the simulations on 50 × 50 × 5 networks, but with the original low-resolution 20 × 20 pixel image stimuli as inputs. Each pixel in the 20 × 20 images was replicated once to create images of 40 × 40. One column and one row of void pixels were then added to the right and bottom of the matrices to make images of dimension 50 × 50. When these low-resolution 50 × 50 stimuli were presented to the networks, the accuracy of familiarity detection remained at 96.7% (Fig. 4*A*), suggesting that increased network dimension was responsible for the accuracy improvement.

Interclass learning of three image classes (car + dog + human) using 50 × 50 × 5 networks also produced stable and accurate results (data not shown). For multiple images, we presented each image for 15 s and then moved to the next image. In contrast, we also tried looping through all images 15 times. The first presentation protocol resulted in slightly better accuracy, and was used for all the remaining simulations.

To further evaluate the limits of these neural networks on multi-face recognition, we converted a database of 200 human face images to 50 × 50 pixel stimuli. In each simulation, a randomly-generated 50 × 50 × 5 network was presented with 10 human face images, each for 6 s, and testing was conducted with the 10 learned faces plus 10 novel faces drawn randomly from the database. We used 6 s instead of 15, to prevent overtraining of the network. After sorting the network firing rate for all 20 faces, a hypothetical threshold was drawn between the responses to faces ranking 10th and 11th, to evaluate the ability of the networks to separate familiar and novel faces. This procedure is similar to the empirical ranking theory of vision (Purves et al., 2011), which suggests that subjects perceive the relative color and brightness of objects by internally ranking the empirical brain activity they evoke. In our case, the percentage of learned faces appearing in the top 10 reflects familiarity detection accuracy. The 10 faces used for presentation to the networks were randomly selected from the database, indexed as 1–10 and fixed for all simulations. The 10 novel faces were randomly drawn from the remaining database and were varied for each testing trial. Figure 4*B* shows the sorted responses and hypothetical threshold for a network that was able to detect 9 out of the 10 presented faces in one testing trial, and therefore was considered 90% accurate for this trial. The average accuracy of a network was measured by averaging the results of 1000 testing trials. Performance of ten randomly-generated 50 × 50 × 5 networks for the above simulations is shown in Figure 4*C*, blue. The best network reached ∼85% accuracy on average for 10-face familiarity detection.

In an attempt to further improve the performance, we generated a different set of ten randomly-generated networks, with one more layer added to the network reservoir (50 × 50 × 6), and increased the connection probability from the input layer to the reservoir (Cscale = 0.004 to Cscale = 0.005). The resulting network average accuracy is shown in Figure 4*C*, red. Not only did the overall accuracy improve, but also a network with >95% accuracy emerged, indicating the network was able to detect all 10 familiar faces accurately for at least half of the 1000 testing trials. Figure 4*D* summarizes the accuracy distribution of 1000 testing trials for the ten 50 × 50 × 6 networks. The distribution is significantly shifted from what would be expected by chance alone, i.e., five familiar faces falling in the top 10. Figure 4*E* plots the response of the best-performing 50 × 50 × 6 network for one of the testing trials. Unsupervised learning has clearly modified the network response and a hypothetical threshold can be drawn which accurately differentiates familiar from novel faces.

We also evaluated the performance of ten randomly-generated 50 × 50 × 5 networks with Cscale 0.005 and ten randomly-generated 50 × 50 × 6 networks with Cscale 0.004 (Fig. 4*C*, dotted lines). Neither achieved comparable accuracy as 50 × 50 × 6 networks with Cscale 0.005. From these results, it seems that both the network size and the number of input connections are important. An increase of input connections together with a larger network results in larger available network space for better familiarity storage.

In fact, the network capacity of multi-face recognition is not restricted to 10 faces. In Figure 4*F*, we plot the accuracy of the ten 50 × 50 × 6 networks after learning 10 faces (red), 30 faces (green), and 60 faces (blue), as well as the chance level for 60 faces (gray). To our surprise, only two networks performed equivalently to or below their corresponding chance levels after learning 60 faces. The remaining eight networks performed significantly better than chance levels (Table 2, *n* = 1000, *p* < 0.01). Accuracy after learning 30 and 60 faces was measured by the percentage of familiar stimuli appearing above the hypothetical threshold (set as below 50% of the population) after pooling the same number of novel stimuli with familiar stimuli. The chance level at 60 faces was calculated by the percentage of familiar stimuli appearing above the hypothetical threshold after sorting network baseline firing rates to the familiar and novel stimuli.

**Table 2.** Statistical table

Network	Data structure	Type of test	*p* value
1	Normality test: failed (*p* < 0.05)	Mann-Whitney rank sum test (one-side)	5.29e^−315^
2	Normality test: failed (*p* < 0.05)	Mann-Whitney rank sum test (one-side)	3.45e^−322^
3	Normality test: failed (*p* < 0.05)	Mann-Whitney rank sum test (one-side)	0.00
4	Normality test: failed (*p* < 0.05)	Mann-Whitney rank sum test (one-side)	0.00
5	Normality test: failed (*p* < 0.05)	Mann-Whitney rank sum test (one-side)	0.03
6	Normality test: failed (*p* < 0.05)	Mann-Whitney rank sum test (one-side)	5.16e^−158^
7	Normality test: failed (*p* < 0.05)	Mann-Whitney rank sum test (one-side)	2.42e^−322^
8	Normality test: failed (*p* < 0.05)	Mann-Whitney rank sum test (one-side)	3.71e^−91^
9	Normality test: failed (*p* < 0.05)	Mann-Whitney rank sum test (one-side)	1.49e^−140^
10	Normality test: failed (*p* < 0.05)	Mann-Whitney rank sum test (one-side)	1.00

In addition, we generated scrambled versions of the 50 × 50 face images from the same database by relocating all pixels to new random (*x*, *y*) positions, and recorded the network responses to them after learning 10 human faces. The results show that networks not only discriminated the familiar faces from the novel faces, but also could discern novel faces from their scrambled versions (Fig. 4*G*). The trained networks appeared to have acquired the concept of a “face” and responded less to scrambled faces.

### Synaptic weight change

**Figure 5.**
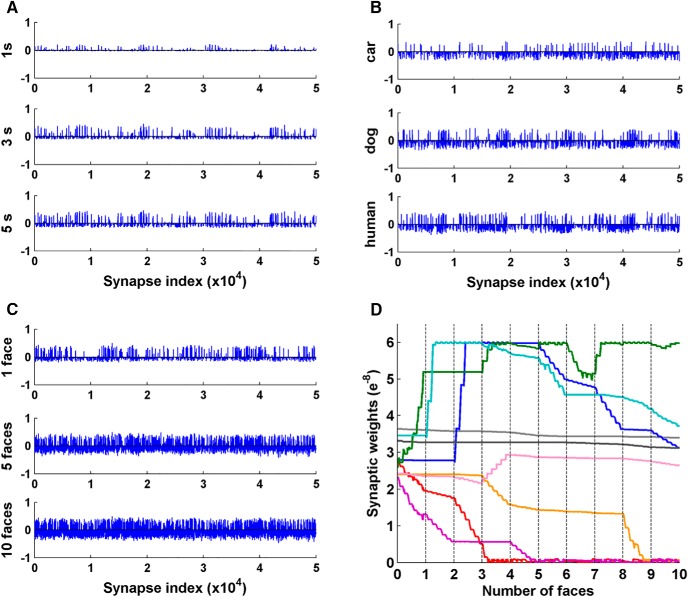
Synaptic weight changes caused by learning. ***A***, Network synaptic weight change after the presentation of 1, 3, and 5 s of the same human face stimulus, in comparison to initial synaptic weights. ***B***, Network synaptic weight change after learning a car, dog, or human stimulus, each for 15 s, in comparison to initial synaptic weights. ***C***, Network synaptic weight change after learning 1, 5, and 10 human face image stimuli, each for 6 s, in comparison to initial synaptic weights. ***A***–***C***, Only a subset of NMDAR synapses in the network are shown for clarity and the *y*-axis scales are ±1e^−7^. ***D***, Time course of weight change of nine randomly-selected NMDAR synapses during the exposure to ten faces, each for 6 s.

Given that synaptic weight changes underlie the observed results described above, we plot in Figure 5 the network weight changes in three scenarios: weight change after exposure to a human face stimulus for 1, 3, and 5 s (Fig. 5*A*); weight change after exposure to a car, a dog or a human face image each for 15 s (Fig. 5*B*); and weight change after exposure to multiple face images (Fig. 5*C*). A common finding emerges for all three scenarios: unsupervised learning caused a subset of synapses to be potentiated, and a different but larger subset of synapses to be depressed. The amplitude of potentiation is larger than the amplitude of depression, on average. This is in line with a proposed familiarization mechanism that a small subset of neurons becomes strongly responsive after sensory exposure while depression occurs pervasively among other neurons, sharpening the familiarity response (Freedman et al., 2006; Meyer et al., 2014). Figure 5*D* shows the time course of the change in synaptic strength for nine representative synapses randomly picked from a 50 × 50 × 6 network during the exposure to 10 faces. Synaptic weights changed in unique ways in response to each face stimulus.

### Subnetwork formation

**Figure 6.**
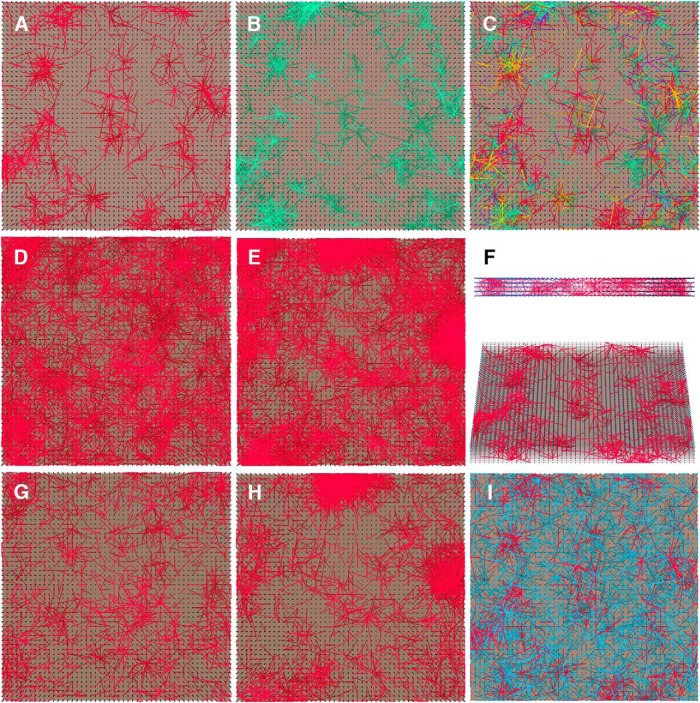
Importance of subnetwork topology. ***A–C***, Colored lines indicate potentiated synaptic connections of a 50 × 50 × 6 network after learning face 1 (***A***), face 2 (***B***), or face 1 and 2 (***C***). Yellow highlights the synapses in ***C*** that also appeared in both ***A*** and ***B***; red and green highlights the synapses that only appeared in ***A*** and ***B*,** respectively. Purple indicates the synapses that newly appeared after learning both faces. ***D***, ***E***, Potentiated synaptic connections in the networks with the best (***D***) and worst (***E***) accuracy after learning 60 faces. ***F***, Side views of the potentiated synaptic connections in ***A***. ***G***, Potentiated synaptic connections of the network in ***D*** after learning 60 faces, in comparison to synaptic weights obtained after learning 30 faces. ***H***, Potentiated synaptic connections of the network in ***E*** after learning 60 faces, in comparison to synaptic weights obtained after learning 30 faces. ***I***, Potentiated (red) and depressed (blue) synapses after learning face 1, for the same simulation shown in ***A***. Thresholds for significant weight increase and decrease are 1e^−8^ and −1e^−8^ for all panels.

If we put together the phenomena of how synaptic weights change after sensory exposure and how available network space affects accuracy, a theory emerges that could possibly address the stability-plasticity dilemma (Grossberg, 1980, 2013; Mermillod et al., 2013). Whenever a new image stimulus enters the network reservoir, an image-representative subnetwork is formed by potentiated and depressed synapses. Providing the network reservoir is large enough and has sufficient complexity, a large number of image-representative subnetworks that differ slightly from each other can co-exist in the network. This reduces the chance that formation of a new memory overwrites an older one, allowing each subnetwork to support familiarity detection. To provide support for this idea, we identified the set of synapses whose weights were modified after the stimulus presentation. In Figure 6*A*,*B*, the potentiated synapses are highlighted in red and green, respectively, for the same network after exposure to two different face images. In Figure 6*C*, the potentiated synapses are highlighted after the same network was exposed to both face images. Notice the difference between the two image-representative subnetworks, and how they can co-exist in the network reservoir. These subnetworks may act as neural clusters carrying memory traces (Liu et al., 2012). In Figure 6*A–E*, the subnetworks are viewed from the top. As the faces resemble each other to a certain degree, the corresponding subnetworks are proximate to each other. They are also densely connected through the layers, as seen in a side view (Fig. 6*F*).

Following this idea, we plotted the potentiated synapses of the ten 50 × 50 × 6 networks after learning multiple faces. Figure 6*D*,*E* shows the potentiated synapses in the two networks with the best and the worst accuracy, after exposure to 60 faces (Fig. 4*F*, network 3 and 10, respectively). Dense clusters can be seen in the poorly-performing network (Fig. 6*E*). We also investigated the evolution of subnetworks from 30- to 60-face exposures. Figure 6*G*,*H* graphs the incremented subnetworks corresponding to D and E. Newly potentiated synapses are spread throughout the reservoir in the network with high accuracy, whereas newly potentiated synapses are clustered in the network with low accuracy. We calculated the average clustering coefficients (Watts and Strogatz, 1998) of the formed subnetworks for the 10 networks after learning 10, 30, and 60 faces, and correlated the average clustering coefficients with the corresponding network accuracies. The data suggest that strong clustering of subnetworks negatively affects network performance (*r* = −0.80, *p* = 9.05e^−8^). In general, subnetworks were more distributed in networks with better accuracy, and more aggregated in networks with poorer accuracy. One possible explanation is that overlapping subnetworks lead to increased chance for existing memories to be overwritten. The dense clustering observed in poorly performing networks might be caused by local densely-connected neurons, which tend to self-potentiate excessively due to recurrent pathways, thereby reducing memory capacity.

For the network with the best accuracy, we attempted to permute the initial weights of all NMDAR synapses of the network and repeated the same simulation of the 60-face exposure. After permutation, the network accuracy failed to remain the best. It seems that the preimposed network circuitry, which is determined by the initial synaptic connection weights, is another important factor for network performance.

### Separability

In addition to using network firing rate as a readout for familiarity detection, we applied Fisher’s discriminant analysis to search for other features that may help define familiarity. Ten 20 × 20 × 5 networks and the single face recognition simulations were used for the analysis. Network response to the familiar stimulus is referred to as the familiar response, and responses to the remaining 29 stimuli are referred to as control responses. FDR is calculated between two classes (see Materials and Methods). Specifically, familiar FDR is calculated by labeling the familiar response as Class I and the control responses as Class II. Control FDRs are calculated by labeling one of the control responses as Class I and the remaining control responses together with the familiar response as Class II. Baseline FDRs are calculated in the same way but with network baseline responses to all 30 image stimuli. Baseline FDR values represent how well each image stimulus is separated from others, before learning.

**Figure 7.**
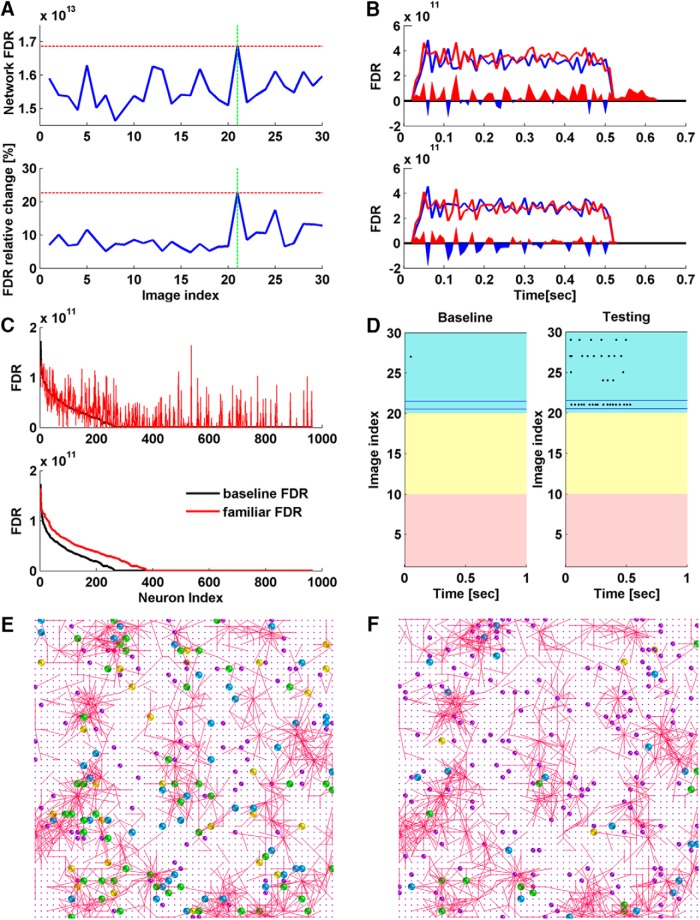
FDR analysis. ***A–D***, FDRs were calculated for the 20 × 20 × 5 network after the single face recognition simulation shown in Figure 3*D*. The input stimulus was human face 1, indexed as image 21 in the set of 30 test images. ***A***, top, Network FDRs after learning human face 1. Bottom, Network FDR relative changes from baseline after learning human face 1. Red dashed lines represent the maximum in the group. Green dashed lines represent the presented stimulus. Network FDR has the largest value to the familiar stimulus after learning, and the largest relative increase from baseline. ***B***, Network FDRs calculated with network activity in 10-ms time bins. Red lines show the FDRs to the familiar stimulus (image 21) and blue lines show the FDRs to one of the control stimuli (image 9). The filled area shows the difference between the two, with red indicating a larger value of the red line and blue indicating a larger value of the blue line. Top, The FDRs calculated based on network response after sensory exposure. Bottom, the FDRs calculated based on network baseline response. ***C***, Neuronal familiar FDRs were calculated for the active neurons (recorded spikes > 0) after the same simulation. Top, Black indicates the sorted baseline neuronal FDRs, and red indicates the familiar FDRs of the corresponding neurons after the simulation. Bottom, black indicates the sorted baseline neuronal FDRs, and red indicates the sorted neuronal familiar FDRs. ***D***, Baseline and testing responses to all stimuli of the neuron with the largest FDR increase in ***C***. Red, yellow, and blue indicate responses to car, dog, and human stimuli. Neuronal baseline response is negligible, indicating an inactive role in the network. After sensory exposure, neuronal firing rate to image 21 was greatly increased. The response also generalized to other human face images (index 21–30). ***E***, ***F***, FDRs were also calculated for neurons in the 50 × 50 × 6 network after the simulation shown in Figure 6*A*. The input stimulus was human face 1 from the 200-face database. The subnetwork in Figure 6*A* connects 1728 unique neurons, including 187 presynaptic neurons, 1238 postsynaptic neurons, and 303 neurons that are both pre- and postsynaptic. ***E***, Intersection of top 200 FDR neurons with the formed subnetwork (*n* = 118). Yellow, blue, and green indicate those FDR neurons that overlapped with presynaptic neurons (*n* = 26), postsynaptic neurons (*n* = 43), and both pre- and postsynaptic neurons (*n* = 49) in the subnetwork. Purple indicates nonoverlapped FDR neurons (*n* = 82). ***F***, Intersection of top 200 FDR neurons calculated for control face 2 with the formed subnetwork after the network was exposed to face 1 (*n* = 37). Yellow, blue, and green indicate those FDR neurons that overlapped with presynaptic neurons (*n* = 4), postsynaptic neurons (*n* = 21), and both pre- and postsynaptic neurons (*n* = 12) in the subnetwork. Purple indicates nonoverlapped FDR neurons (*n* = 163).

Compared with the corresponding baseline FDRs, we see a general increase for both familiar FDR and control FDRs after sensory exposure (Fig. 7*A*). In 26 out of 30 cases, familiar FDRs show the largest magnitude of relative increase from baseline compared with control FDRs; and in 22 out of 30 cases, familiar FDRs have the largest final values. Larger familiar FDRs indicates that the network discriminated the familiar better from the control stimuli after learning, implying greater separability at the network level. Figure 7*B* shows the network FDRs calculated in each time bin. The larger familiar FDR can be explained by the larger FDR values in each time bin. Sensory exposure has also extended the network separability to beyond the stimulus presentation window (0–0.5 s).

To further understand the changes occurring at the neuronal level that supported familiarity detection, we applied the analysis to individual neurons. Similarly, familiar FDR is calculated by labeling the neuronal response to the familiar stimulus as Class I and responses to control stimuli as Class II; control FDRs are calculated by labeling the neuronal response to one of the control stimuli as Class I and responses to the remaining control stimuli and the familiar stimulus as Class II.

Compared with corresponding baseline FDRs, neurons that show increased FDRs to the familiar stimulus are considered as critical and potentially correlate with the emergence of familiarity. We noticed that the neurons with significantly increased FDRs tended to evolve more often from the neurons with negligible baseline FDRs (Fig. 7*C*). Neuronal baseline FDR was found to be negatively correlated with FDR increase (*r* = −0.30, *p* = 4.46e^−164^). The top-ranking neurons showed little response at baseline but increased firing rate after sensory exposure. The low baseline response implies that they did not receive much information from the input layer initially, or equivalently, that they were not wired to the input-responding pathway before the training. The increase in firing rate after learning implies that their connections to the input-responding pathway were strengthened. These critical neurons are recruited by unsupervised learning to the subnetwork that responds to a specific stimulus. Once recruited, they may also fire to other nonlearned inputs, but their response to the familiar input is stronger (Fig. 7*D*).

So far, we have looked at the formation of subnetworks (altered connections) and the emergence of critical neurons for familiarity detection. The intersection between the two intrigues us. Therefore, we investigated whether the critical neurons belong to the subnetworks. The subnetwork in Figure 6*A* was analyzed. We selected 200 critical neurons with top-ranking familiar FDR values and 118 of them belong to the subnetwork (Fig. 7*E*). If 200 neurons were randomly selected from the network reservoir, we would expect ∼28 neurons to overlap with the subnetwork by chance. For comparison, we also selected 200 critical neurons with top-ranking control FDR values for control faces 2–10 for the same network, after learning face 1. The intersections with the subnetwork dropped to 43 ± 8 (mean ± SD) neurons (Fig. 7*F*). The observation held when other faces were used for learning. Therefore, the critical neurons selected for the learned face and the subnetwork formed after exposure are highly correlated. Their colocalization in space further supports the idea that the potentiated subnetwork is an important memory storage unit.

### Effect of STDP and LTD/LTP

**Figure 8.**
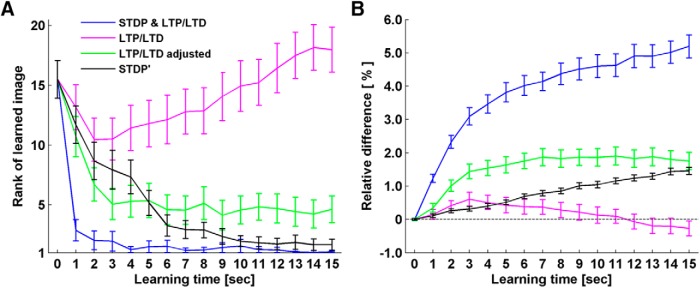
Impact of STDP and LTP/LTD on familiarity development. ***A***, Rank of network firing rate for the familiar image among 30 testing images is plotted as a function of learning time. The initial rank before learning is 15th on average, while an end rank of 1st indicates the image is detected as familiar. Single face recognition simulations were repeated with full NMDAR plasticity (STDP and LTP/LTD); without STDP after removing BPAPs (LTP/LTD); without STDP but after retuning the calcium control hypothesis (LTP/LTD adjusted); and STDP plasticity alone (STDP’). Error bars represent the SEM of 30 simulations conducted on 10 networks. ***B***, Relative difference in network firing rate to familiar and novel inputs after learning with different forms of plasticity. The relative difference is calculated as the discrepancy between network response to familiar input and the average of network responses to novel inputs, divided by the response to the familiar input after learning. Same simulations and same color code as in ***A***.

As the NMDAR-based plasticity we implemented supports both LTD/LTP and STDP mechanisms, we investigated how they contributed to familiarity detection. Learning in which LTP/LTD and STDP worked together stabilized network performance efficiently to the optimum accuracy (Fig. 8*A*, STDP & LTP/LTD). Then we selectively deactivated STDP by removing BPAPs (Eq. 4) and repeated the single face recognition simulations on 10 networks. The results showed a severe reduction of accuracy (Fig. 8*A*, LTP/LTD). At later time points during learning without STDP, network accuracy reversed and networks responded less to the familiar input than to novel inputs on average. This is the result of an LTD-dominant Ω function (Eq. 10) tuned to account for the effect of BPAPs. To know what LTP/LTD alone is capable of, we need to readjust the parameters of the Ω function to compensate for the loss of BPAPs (Fig. 8*A*, LTP/LTD adjusted, $\alpha_{1}=0.3,\alpha_{2}=0.4\,\;and\,\;\Omega_{rate}=0.3$). Comparing the curves of STDP & LTP/LTD, and LTP/LTD adjusted (Fig. 8*A*), we postulate that the effect of STDP is to increase learning specificity.

Does this mean that STDP alone could be sufficient for familiarity detection? As STDP cannot be isolated to function alone under the calcium control hypothesis, we repeated the single face recognition simulations using the STDP synapse model available in the CSIM package, which is based on the work by Froemke and Dan (2002) and Gutig et al. (2003). We adopted the same parameter values used in Song et al. (2000) and Xue et al. (2013). For comparison, we used the same 10 networks, replacing the NMDAR synapses with STDP synapses. Initial synaptic weights were kept the same to ensure that the networks’ baseline responses to the input stimuli were comparable to the previous values obtained with NMDAR synapses. Then we switched on the STDP plasticity and repeated the single face recognition simulations (Fig. 8*A*, STDP’) . Learning accuracy of networks with STDP-only synapses improved gradually, but it did not reach the optimum value (rank 1) on average after 15 s of learning. The NMDAR model which incorporates both STDP and LTP/LTD was more accurate and more efficient. The network response relative difference after learning with STDP-only synapses was also much smaller than with NMDAR synapses (Fig. 8*B*).

Note that the STDP mechanism, which is part of the NMDAR synapses, works through calcium concentration, whereas the STDP synapse in CSIM works through an equation which uses the time intervals between presynaptic spikes and postsynaptic spikes as inputs. On average, STDP-only plasticity requires repetitive presentation of the stimulus to develop accurate familiarity. While with NMDAR-dependent plasticity, networks develop familiarity fast and efficiently, as required for one-shot learning (Yakovlev et al., 2008). Therefore, it seems that the calcium control hypothesis is an efficient way of combining the frequency rule (LTP/LTD) and the timing rule (STDP), enhancing both forms of plasticity.

### Up- and down-states, bursting, and background noise

It has been suggested that being at the “edge of chaos” (Crutchfield and Young, 1990; Langton, 1990) is desirable for neural networks performing complex computational tasks (Bertschinger and Natschläger, 2004). Networks functioning in regimes that are either too chaotic or too ordered in their response to input stimuli do not perform well on computational tasks. Legenstein and Maass (2007) tested the impact of the parameters Wscale and λ in CSIM (see Materials and Methods) for network performance on certain classification tasks. Wscale controls the strength of synaptic connections in CSIM, while λ controls the number and average distance of synaptically connected neurons. The values of Wscale (0.9) and λ (4) that we used in the NMDAR-LSM model were determined empirically using network performance in the familiarity-novelty tests, and they happen to be at the transitional boundary, (between ordered and chaotic responses) identified by Legenstein and Maass, an area that was reported to have a good trade-off between accuracy and generalization.

**Figure 9.**
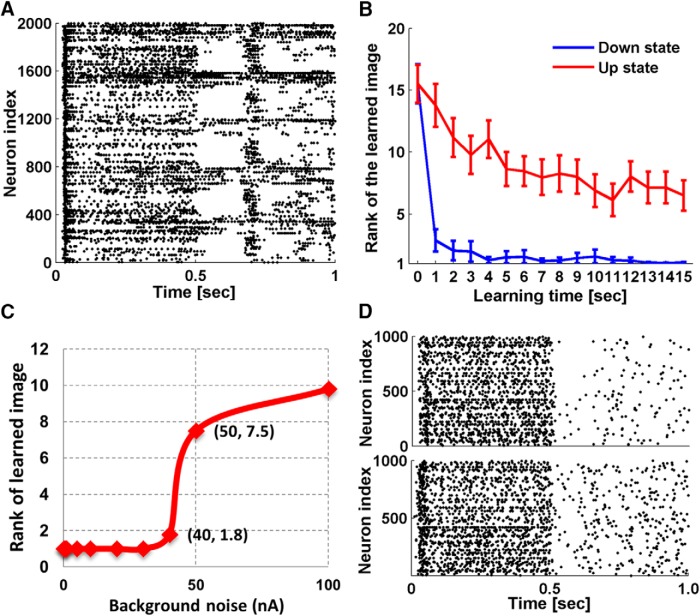
Effect of bursting and noise. ***A***, Response of a 20 × 20 × 5 up-state network after learning a human face image for 11 s. A network burst spontaneously emerged in the interval of 0.5–1.0 s when no input stimulus was given. ***B***, Impact of bursting on network performance. Rank of network firing rate to the familiar image among 30 testing images is plotted as a function of learning time. Blue represents simulations with down-state networks, the same networks as we used in the single face recognition simulations. Red represents simulations with up-state networks (Wscale was set tenfold of the original value used in simulation). Error bars are the SEM of 90 simulations conducted on 30 networks. ***C***, Impact of background noise on network performance. Average rank of network firing rate to the familiar image among 30 test images after learning for 15 s is plotted as a function of background noise. ***D***, Network spiking activity of a 20 × 20 × 5 network to the input stimulus, simulated with 40- and 50-nA background noise, respectively.

Legenstein and Maass also identified the existence of up and down-states (Cowan and Wilson, 1994) in their simulated neural network models. These states refer to dynamic regimes observed in intracellular recordings of mammalian central nervous system neurons that differ in their membrane potential and conductance properties. In the down-states, the membrane potential is hyperpolarized and stable and membrane conductance is low. In the up-states, the membrane potential is more depolarized, highly variable and membrane conductance is high, resulting from a continuous bombardment of background synaptic inputs. In neural networks, up-states are characterized by network-wide synchronized bursting activity (Johnson and Buonomano, 2007). As no bursting was observed in our simulations, it seems that the tuned Wscale and λ parameters constrained the networks to be in down-states. To know how bursting would affect the network performance, we removed the biological boundaries of the parameters. In one simulation, we increased Wscale tenfold while preserving λ. Bursting emerged during stimulus presentations (Fig. 9*A*), and we measured network accuracy through single face recognition simulations and compared the results with the results described above (Fig. 9*B*). It turns out that in a bursting network, network performance is compromised, and more learning is required for comparable accuracy. Bursting also emerged with a twofold increase in λ while preserving Wscale, which induced similar impairments of network accuracy.

Network accuracy is also affected by the amount of background noise current injected into the neurons (Eq. 1) during simulation. We measured network accuracy through single face recognition simulations with background noise ranging from 1 to 100 nA. Figure 9*C* shows that network accuracy is preserved until the noise exceeds 40 nA, which is unrealistically high compared with normal ranges used in CSIM. A dramatic performance decline is seen from 40 to 50 nA, and the corresponding network responses are exemplified in Figure 9*D*. Background noise of 50 nA clearly introduced more random spikes during simulation. If the number of random spikes exceeds the discrepancy of firing rate that was previously used to differentiate familiarity from novelty, it is very likely to disrupt network performance.

## Discussion

In the simulations described above, we have investigated the ability of recurrent spiking neural networks supplemented with NMDAR plasticity to detect familiar sensory inputs following unsupervised learning. Unsupervised learning is biologically plausible and has been shown to be critical for the development of the mammalian central nervous system, for example, in the visual pathway (Blasdel et al., 1977). In the beard versus no-beard classification simulations, our neural networks learned to differentiate “beard” and “no-beard” face images. The single face recognition simulations were conducted with a more rigorous design to investigate the specificity of familiarity detection. Networks of 20 × 20 × 5 neurons connected by NMDAR-containing synapses were able to detect a previously-seen face accurately in most of the simulations, with a pronounced and specific increase in overall firing rate for the familiar stimulus. Responses to the control groups did not deviate far from baseline.

Our simulations with larger networks (up to 50 × 50 × 6 neurons) indicated that multiple familiarity traces can be learned and stored. The storage capacity increased with available network space. Our largest network model (15,000 neurons) can store familiarity traces for 60 faces, before accuracy drops to chance level. One possible explanation is that within large networks, subsets of synaptic connections that are selectively potentiated or depressed for each face can co-exist, reducing the chance of a new memory overwriting an older one. This observation suggests a potential resolution for the stability-plasticity dilemma. Considering there are billions of neurons in the human brain organized in a very large number of microcircuits, it seems reasonable, in principal, that human recognition memory has an apparently limitless capacity, as reported by Standing’s experiments (1973). Nevertheless, it is important to mention that in Standing’s tests, a two-alternative forced-choice paradigm was used. In a subsequent study that supported Standing’s finding on memory capacity (Brady et al., 2008), the two-choice paradigm was also used. A higher rate of recall error was reported by Laeng et al. (2007), when conducting similar image recognition experiments using a yes-no paradigm with only one picture presented. Whether the two-choice paradigm is justified as a good measure for recognition and a good indication for memory capacity is arguable (Laeng et al., 2007). Our analysis relied on a comparative paradigm similar to Standing’s two-alternative tests: to interpret the data and measure familiarity, a threshold needs to be set for the network firing rate, which was done empirically comparing familiar and novel responses. Once the threshold is set, the networks can also make yes-no decisions for individual inputs (Fig. 4*G*).

In addition to the specificity and capacity for familiarity detection, we also noticed a generalization effect in the responses of the trained networks to input stimuli. Evidence for generalization was observed when, following training, the response to novel input stimuli was increased selectively for those that belonged to the same class as the trained inputs. In the beard versus no-beard classification simulations, generalization was observed when the trained networks were able to distinguish novel beard from novel no-beard images. In single face recognition simulations, the networks generalized familiarity response to a subset of images from the same class as the presented stimulus. In the multi-face recognition simulations, the networks apparently learned the concept face after training on ten face images: they responded stronger to novel faces than their scrambled versions. Recall that for 10 × 10 × 5 networks, input neurons were set to form synapses one-to-one with the first layer of the network reservoir, whereas for networks of 20 × 20 × 5 or larger dimension, input neurons were set to form synapses randomly with the network reservoir by probability. Innervation by probability is a better way to use the network space and prevents overtraining of the first layer of the network reservoir. Meanwhile it disrupts the spatial patterns present in the face images, making the familiarity detection a high-dimensional challenge. Yet the generalization property remained with the randomly projected inputs, which speaks to the robustness and computational power of our networks, i.e., learning specificity was preserved while generalization was acquired. When multiple inputs from a class were learned, generalization allowed the networks to act as an unsupervised classifier, by automatically classifying input stimuli based on past sensory experience. In contrast, conventional classifiers require properly labeled data of all classes to be trained.

Fisher’s discriminant analyses suggest that familiarization occurred in high-dimensional feature space. Network connections were modified and neurons in hidden circuits were recruited to respond to familiar inputs. Neurons hidden from the input layer are analogous to neurons from deep layers of a feed-forward network, or neurons from the downstream circuits in higher-order brain regions. Their recruitment suggests how signals can be relayed to brain regions other than the primary processing region and cause differences in neuronal activity. As the deep-layer neurons showed unique firing patterns to the familiar stimulus, their activity can be used to identify the input pattern, a function similar to the “grandmother-cells” identified by *in vivo* brain recordings (Quiroga et al., 2005). For decades neuroscientists have debated the possible forms of information encoding in the brain, such as parallel distributed processing versus single neuron firing. Results presented here show they are not mutually exclusive. While a familiar input is encoded in a network of thousands of neurons, it may also selectively activate single neurons in deep layers.

Familiarity studies stemmed from experiments at the behavioral level. Several groups have conducted experiments with the familiarity/novelty paradigm *in vivo*. Studies using fMRI measurements (Kosaka et al., 2003; Gobbini and Haxby, 2006) found an increase in the BOLD signal to familiar stimuli. Recordings in the inferior temporal lobe of behaving monkeys have demonstrated differential responses to novel and familiar images (Anderson et al., 2008). Interestingly, familiar images evoked larger-amplitude local field potentials, whereas multi-unit spiking responses were greater for novel images. Finally, a phenomenon called stimulus-selective response potentiation was identified in rodent visual cortex recordings (Frenkel et al., 2006; Cooke and Bear, 2010; Gavornik and Bear, 2014). It is a form of experience-dependent response enhancement during visual experiments. We think it supports the existence of intrinsic familiarity in the visual cortex.

A few neural network models have been proposed to study network-level learning and memory. Several of them applied STDP to various network architectures. For example, a few groups (Lazar et al., 2007; Oliveri et al., 2007; Xue et al., 2013) investigated LSMs with STDP and the results suggested an enhanced computational capability. In these models, STDP was either applied to train readout neurons or to modulate neuron excitability, rather than allowing it to directly modify synaptic weights in the network. Studies that used neural networks other than LSMs (Clopath et al., 2010; Carlson et al., 2013; Klampfl and Maass, 2013; Zheng et al., 2013; Srinivasa and Cho, 2014) have reported emerging learning and memory after applying STDP to recurrent network architectures. Nevertheless, the networks they used are either of a preimposed wiring diagram (Klampfl and Maass, 2013) or highly simplified (Srinivasa and Cho, 2014), and therefore poorly replicate cortical microcircuits. Furthermore, these studies solely consider STDP for plasticity while we combine the two major forms of plasticity, LTD/LTP (frequency rule) and STDP (timing rule), based on the calcium control hypothesis, rather than phenomenological equations. Evidence for the interplay of LTD/LTP and STDP has been found in the literature, and separating them by firing rate or spike timing might lead to an artificial dichotomy (Sjöström et al., 2001). Our simulation results suggest a mutual-enhancing effect by combining STDP with LTP/LTD, and this could potentially explain one-shot familiarity memory (Yakovlev et al., 2008). Additionally, some of the reported models require homeostatic control (Clopath et al., 2010; Carlson et al., 2013; Zheng et al., 2013) or inhibitory-STDP (Srinivasa and Cho, 2014) to attain network stability, but in our NMDAR-LSM networks, we relied on the intrinsic balance between LTD and LTP instantiated by the calcium control hypothesis for stability.

The plasticity model we implemented was created to model bidirectional synaptic plasticity through NMDARs (Shouval et al., 2002). Whether NMDARs are solely responsible for this form of plasticity is still controversial, as voltage-dependent Ca^2+^ channels (Nevian and Sakmann, 2006) and metabotropic glutamate receptors (Gladding et al., 2009) have been reported to be capable of inducing synaptic plasticity as well. Yet there is growing evidence that bidirectional modifications can be induced through NMDAR-dependent pathways alone (Hunt et al., 2013; Huang et al., 2014). The results presented here demonstrate that bidirectional synaptic plasticity is sufficient to endow neural network models of generic cortical microcircuits with the ability to detect familiar sensory inputs through unsupervised learning. This has important consequences for mammalian brain development, since it suggests that these universal building blocks of the cortex have an inherent ability for familiarity detection.

In NMDAR-LSM networks, recurrent spiking neural networks expand input stimuli into high-dimensional feature space (Maass et al., 2002). Unsupervised learning altered the feature space to allow linear separation of familiar from novel faces, by formation of subnetworks specific for each input stimulus. Learning multiple inputs belonging to a class (e.g., beard, face) resulted in generalization, allowing the network to classify novel input stimuli. This relationship between familiarity detection, generalization, and classification needs to be studied in more depth.

*Note Added in Proof:* The title of the article was incorrectly listed in the Early Release version. The title has now been corrected.

## Synthesis

Reviewing Editor: Tatyana Sharpee, The Salk Institute for Biological Studies Decisions are customarily a result of the Reviewing Editor and the peer reviewers coming together and discussing their recommendations until a consensus is reached. When revisions are invited, a fact-based synthesis statement explaining their decision and outlining what is needed to prepare a revision will be listed below. The following reviewer(s) agreed to reveal their identity: Dean Buonomano.

The manuscript describes how synaptic plasticity within recurrent network can support familiarity detection. The main points to address are to identify essential aspects of the model that subserve familiarity detections, such as whether STDP-like synaptic plasticity, 2D or 1D networks might suffice, as well as identifying key topological features of the network quantitatively (see Reviewer 2 comments). The manuscript could benefit from streamlined presentation (see specific suggestions from reviewers).

Reviewer 1:

The paper provides a fairly simple model of an important and commonly studied cognitive task: face recognition and image familiarity. The model provide testable predictions and should be helpful to cognitive neuroscientists studying recognition memory and familiarity.

This computational paper examines the ability of a spiking recurrent network to recognize (discriminate between familiar/unfamiliar visual images). The networks are composed of excitatory and inhibitory units in which the excitatory synapses are plastic. The general approach is that images such as bearded faces are presented to the network with plasticity on, and then total spike counts in response to bearded faces versus unbearded faces are compared. All decoding is based on network-wide or neuron specific spike counts. The results clearly establish that this approach generates larger network responses to the familiar stimuli. This holds true for both the specific images as well as the general class of images (e.g., faces x cars)-thus the network exhibits interesting generalization properties. The authors explore a number of properties of the network including the role of subnetwork topology and of different forms of synaptic plasticity. Overall the model is based a reservoir network approach, but extends standard reservoir networks by incorporating plasticity. Over this is an interesting study that parallels some imaging studies that look at BOLD responses to familiar and unfamiliar images, and that provides some insights/predictions to the potential mechanisms of this form of memory.

The paper is fairly large as it is and it is probably not justifiable to make it larger. But it would be important to briefly mention the limitation of the readout mechanism used here. While spike counting across the entire network across large windows provides a simple quantification mechanism, effectively using this information can be tricky if the average firing rate of the network changes over stimulus presentation time. That is, one cannot simply set a total spike threshold at X if X is always exceeded at the onset of the stimulus. Figures 9 and 11 did show us the performance across time bins, but did not reveal if the average firing rate is changing during the stimulus. On a related note, in the neuron based FDR analyses, does the FDR of the high information neurons change over time bins? I recommend adding a bin-dependent progression of neuron-specific FDR to Figure 11 (even if only for a few individual neurons)-which will provide the reader with a bit of insight to whether the code is dynamics or not.

The title states the importance of NMDA-dependent plasticity-but actually the model emphasizes the importance of STDP-like plasticity as well, and it is far from clear if the LTD component of STDP is NMDA-dependent. Although I understand the implementation is based on previous NMDA-dependent bidirectional models of plasticity, I assume the conclusion of the paper is that bidirectional associative plasticity is key whether or not it is NMDA-dependent.

Pg. 23 & Figure 10. What happens with just STDP?

The paper has a lot of figures, and it may be possible to decrease their number. For example Figs. 3 and 4 can be merged or one of them deleted.

The authors cite Standing 1973 for evidence of our large (“imitless”) capacity for recognition memory. But it is important to point out that Standing's study is controversial (e.g., Laeng et al, 2007, Brain & Cognition).

Line 215: “count of at time”

Line 238: “bear or no-beard”

Reviewer 2:

This paper presents a 3D network with several layers of excitatory and inhibitory neurons that are connected to each other with plastic neurons. The authors use a standard model on NMDAR dependent plasticity and show that once this plasticity model is included and the network is trained with images, it develops familiarity detection. That is, it responds more strongly to familiar than to novel images. This result is not very surprising, but still novel. However, I have several significant problems with this manuscript.

The first and significant problem is that the authors preform “experiments” on the network (for example line 498). That is, they set up a relatively complex network and run simulations, and as experimentalists they do they characterize the results. The aim of theory is to elucidate mechanisms in a way that cannot be done in experiments. Here they do experiments on an artificial model, without understanding why this model does what it does. Furthermore, the analysis of this network is very qualitative in a way that would not even be sufficient for experiments. In this model we do not really know what causes the familiarity detection. Is it the learning rule is it the complex 3D network are both needed? Why when the size increases, there is a very significant improvement in performance? All of these problems could be addresses even without sophisticated analysis. For example, the authors could start with a 1D fully connected network with plasticity. Would such a network exhibit the same characteristics? If not, what minimal change would improve it etc. As they have done it, they have set up a complex network from the outset and we do not know which features are necessary and which are not. Such a naïve process would not tell us why the network behaves as it does, but it will at least tell us what are the necessary features. However, changing this would be difficult it would require preforming a totally new set of simulations and analysis.

Another problem is that the description of what the authors actually do and present is not clear at all. For example, a basic concept throughout is “network activity” but this is never defined. Many cells in the network are active throughout the presentation of the image. Is network activity simply the sum of spikes during this time period? If this is the case, what are the dynamics of the network activity? Also, what are the statistics of the network activity? There is some randomness to the network as there is a noise term that is (sometimes?) used, and there is randomness to the reset. Is the response variability similar to what we would expect experimentally? Additionally, how is “familiarity detection” defined? It is quantified but its mathematical definition is still not clear to me. This problem should be easy to fix.

Another problem is that the experimental results cited for familiarity detection are primarily behavioral, but the simulations show differences in the simulated physiology. Do we know experimentally what changes such a training protocol would cause to real cortical networks? If we do this should be the target, not behavior which is still many steps away from the initial response of the sensory cortex.

Details comments:

Figure 2-4 are not necessary, this has been previously shown and done in published work. Possibly compress all of these into a single image.

Fig 5A. Dynamics are shown for a limited amount of time. Does the change reach a steady state, or would the difference keep increasing? Also why start with the difference, it would be more honest to start with the raw activity changes and only then show the difference. Indeed, when we look at the subsequent subplots (note where the y axis scale starts) the relative difference is very small, on the order of 10%. It would be more honest to show the relative difference. Going back to the first point though, does this difference increase over time, blow up or go down again.

Still with figure 5 - I would try to show something about the dynamics of the network activity before anything else. A simple option is to show raster plots of a randomly selected set of neurons before and after training. Also show how network activity (whatever that is) changes within the presentation of a single stimulus. Is the response constant, is there a rising phase, a falling phase, etc.

Why do we need the 3D networks, do they give us anything a 2D network would not?

In the multi face case the authors (283) talk about recognition. Is this recognition or still familiarity detection?

Figure 6A,B

Changes seem large in 6A just because rank is used. Actual changes in activity as seen in 6B are small.

Figure 6G - this primarily shows that the responses to all faces grow and the relative difference in growth between familiar and novel is much smaller than the total magnitude of change. This is the same for the previous case as well. However, it is not clear to me if this growth for example depends on the initial conditions? IF one started with stronger initial efficacies, would this growth still be observed? This also relates to the first problem. I suspect that as for most Hebbian rules this network when trained with images will not have a stable fixed point, and will suffer from the standard positive feedback loop - resulting in runaway activity.

Figure 7. It would be interesting to see how weight distributions change over time during training.

Line 382 - “In general, subnetworks appear to be more distributed in networks with better accuracy, and more aggregated in networks with poorer accuracy.” This is a very qualitative statement - even experimentalists could not get away with such a statement without showin proof. What they do show is at best anecdotal.

Figure 9A should really be part of the analysis of figure 5. Also note the small quantitative change in FDR.

Line 425 - “We notice that as the number of presentations increased during learning, the neurons with significantly increased FDR tended to evolve more often from the neurons with negligible baseline FDRs (Figure 9C).” Again, this is qualitative and anecdotal (9C). such statements must be quantified or dropped.

Line 449 - after removing the BPAP plasticity in general will be much weaker, this effects not only STDP. How do we know what is the specific effect of STDP on these results? An alternative will be to keep exactly the same learning rule, but use for training jittered spike trains (for example each spike is randomly jittered by plus or minus 30 ms). This way the detailed information about spike times is lost, but rates are preserved.
